# Could cardiac autonomic modulation be an objective method to identify hypobaric hypoxia symptoms at 25.000ft among Brazilian military airmen?

**DOI:** 10.3389/fphys.2022.1005016

**Published:** 2022-11-03

**Authors:** Fernando Sousa Honorato, Lysleine Alves de Deus, Andrea Lucena Reis, Rodrigo Vanerson Passos Neves, Hugo de Luca Corrêa, Ana Paola Brasil Medeiros, Débora Fernanda Haberland, Radamés Maciel Vitor Medeiros, Jonato Prestes, Carlos Ernesto Santos Ferreira, Thiago Santos Rosa

**Affiliations:** ^1^ Graduate Program in Physical Education, Catholic University of Brasília—DF, Brasília, Brazil; ^2^ Aerospace Medicine Institute—Brazil, Rio de Janeiro, Brazil; ^3^ Physical Education Department , University Center of Rio Grande do Norte — RN, Natal, Brazil

**Keywords:** hypoxia, altitude, aerospace medicine, physical exercise, heart rate variability, flight safety

## Abstract

Hypobaric hypoxia during a flight can cause accidents, resulting in deaths. Heart rate variability may be more sensitive than self-reported hypoxia symptoms to the effects of HH. The level of physical fitness can contribute to efficient cardiac autonomic modulation. However, no studies have examined the association between fitness, heart rate variability, and the time of onset of hypobaric hypoxia symptoms. To analyze the influence of hypobaric hypoxia on cardiac autonomic function at the time of onset of the first symptoms and its association with physical fitness. Male airmen trained and belonging to the staff of the Brazilian Air Force (*n* = 23; 30 ± 6.7 years) participated in a flight simulation in a 25.000 ft hypobaric chamber. Heart rate variability was recorded with a Polar^®^ cardiac monitor. Data were analyzed in the time-domain method using Kubios software. We evaluated pulse oximetry with the Mindray PM-60 oximeter. Physical fitness assessment test results were collected from the archive. At moments rest vs. hypoxia revealed a decrease in heart rate variability indices iRR and RMSSD (*p* < 0.001). The individual analysis of hypoxia-rest variation showed that 100% of the airmen had a negative delta for both iRR and RMSSD indices. The time of onset of hypoxia symptoms was not associated with body composition, physical fitness, oxygen saturation, and HRV indices. Also, we suggest that cardiac autonomic modulation seems to be more sensitive to the effects of hypobaric hypoxia at 25.000 ft than the self-reported subjective perception of symptoms. Further devices that alert to a hypoxic condition during a flight should consider heart rate variability allowing more time and security to reestablish control of the flight.

## 1 Introduction

The environmental characteristics of flight (e.g., low partial pressure of oxygen due to high altitude) pose numerous challenges to physiological homeostasis. Bouak et al. (2018). The occurrence of hypobaric hypoxia (HH) is one of the most serious risks during a flight, as its effects can affect the cognitive and psychomotor capacity of the airmen, directly compromising piloting (Rice et al., 2019) and flight performance (Steinman et al., 2017), and increasing the risk of incidents and accidents that can result in death (Files et al., 2005).

The human being submitted to the condition of HH is subject to a reduction in the oxygen (O_2_) offered to corporal tissues by the arterial blood due to the reduction in the partial pressure of O_2_ in the atmospheric air (de Carvalho et al., 2018; Russomano and Castro, 2020). In an attempt to reestablish physiological homeostasis, the autonomic nervous system acts through biofeedback, promoting the physiological adjustments necessary to provide O_2_ to the tissues, such as an increase in heart rate and blood flow (Hawley et al., 2014; Russomano and Castro, 2020).

In this sense, several studies have evaluated the regulation/action of the cardiac autonomic system during HH aiming to guide the best physical preparation and training of airmen so that they can (if necessary) go through this physiological stress without compromising their physical integrity, and ensuring flight safety (Barak et al., 1999; Vigo et al., 2010).

Flight training in a hypobaric hypoxia simulation chamber aims to provide airmen with the perception of the physiological symptoms induced by HH and instructions on risk management for performing the necessary life support procedures, such as: supplementing with O_2_ through the mask, reestablishing pressurization of the aircraft, and/or reducing the altitude of the aircraft (Brazil, 2017). In this sense, methods that objectively report the physiological symptoms can serve as confirmation of these symptoms and alert to the occurrence of HH. A non-invasive instrument, providing simple measurement and handling, easy access, and low cost, which could be an important tool in this process, is heart rate variability (HRV).

Studies have used HRV as a tool to assess cardiac autonomic modulation (Freeman and Chapleau, 2013; Taralov et al., 2015), in addition to being efficient to quantitatively report the adjustments in the cardiac autonomic system induced by HH (Barak et al., 1999; Vigo et al., 2010; Zhang et al., 2014; Zhang et al., 2015). It is important to emphasize that the manifestation of these physiological adjustments varies according to the individual and, mainly, with the severity of the hypoxia, that is, exposure time and elevation of altitude (de Carvalho, 2015; Russomano and Castro, 2020).

Furthermore, in hypoxia flight simulations in the hypobaric chamber, the time of useful consciousness (TUC) was studied, that is, the time lapse between the loss of O_2_ and the maintenance of the user awareness of the airmen inserted in this environment (Izraeli et al., 1988; Yoneda et al., 2000), commonly assessed by cognitive methods (Rice et al., 2019). Nonetheless, considering the risks to airmen’s health and physical integrity and the fact that this training aims to experience hypobaric hypoxia symptoms, TUC was replaced. Nowadays, to guarantee the physical integrity of the airmen, the time of onset of symptoms (TAS) is currently used in training in HH, that is, the time lapse between the loss of the O_2_ supply and the moment when the airman notices the first symptoms of the physiological change induced by HH, and reports them to the instruction team (Brazil, 2017). Among the self-reported perceptions are vision changes, slow thinking, limb tingling, headache, and euphoria (Brazil, 2017).

Thus, considering that the TAS time-lapse is shorter than the TUC (commonly used in studies), that HH induces changes in cardiac autonomic function, and that, to date, studies have verified cardiac autonomic changes only in TUC (Barak et al., 1999; Vigo et al., 2010), it is plausible to infer that HRV could be an instrument for confirming the physiological effects of HH during TAS. This would enable the perception of the first symptoms of HH to be confirmed and quantified through HRV indices, optimizing the reaction time in the resumption of flight safety before the proximity of the stage of loss of useful consciousness.

Another important aspect of the training of airmen in the military environment is physical fitness. Therefore, airmen are encouraged to practice physical exercises and are regularly submitted to the Physical Conditioning Assessment Test (TACF), for which a minimum level of physical fitness is required (Brazil, 2011). Furthermore, it has been shown that good physical fitness is related to good balance in cardiac autonomic activity (De Meersman, 1993). This fact is possibly justified due to the consensus of the scientific literature on the cardioprotective effect and positive influence on cardiac autonomic function conferred by physical exercise (Kwon et al., 2014; Carter and Ray, 2015). The numerous benefits conferred by physical exercise to its practitioners, in general, are unquestionable (Hawley et al., 2014; de Sousa et al., 2017; Simoes et al., 2017; Deus et al., 2019; Correa et al., 2021) and those which can help airmen in the exercise of flight safety, by improving tissue resistance to hypoxia are also highlighted (Brown and Moore, 2007; Lalonde et al., 2015; Quindry, 2017). It is reasonable to infer that physical exercise practitioners will have better physiological conditions of response to stressor events such HH.

Finally, it is conjectured that airmen with better physical fitness and better autonomic balance, quantified by HRV, will withstand the effects of HH for a longer time. However, as far as we know, no studies have verified the relationship between the physical fitness of airmen and the time of resistance to the effects of HH, marked by the appearance of the first symptoms (TAS). Thus, it is extremely important to assess whether the minimum physical fitness required of airmen in the Brazilian Air Force can help them to mitigate the effects of HH on 25.000 ft.

Therefore, the present study aimed to analyze the influence of HH on cardiac autonomic function during TAS, and its association with physical fitness in Brazilian Air Force airmen.

## 2 Methods

The methods and procedures were approved by the Institute of Aerospace Medicine (IMAE), COMAER protocol n^o^ 67442.003040/2019-01 and by the local Research Ethics Committee under CAAE protocol n^o^ 23003519.80000.0029. The methods and procedures were performed according to the guidelines of the Brazilian Air Force Command in the Physiological Adaptation Stage (EAF) (Brazil, 2017).

All procedures were clearly explained to the airmen, and those who met the requirements of the Institute of Aerospace Medicine and who agreed to participate in the study, signed the Consent Term.

### 2.1 Sample

Airmen (*n* = 26) were evaluated for inclusion and exclusion criteria. The inclusion criteria were: 1) members of the Brazilian Air Force who participated in the flight simulation in the hypobaric chamber of the quadrennium corresponding to October and November 2019; 2) airlift pilot; 3) have the health card issued by the Special Health Board valid and without restrictions for air activity; 4) receive a favorable opinion from the physician before starting the flight simulation in the hypobaric chamber; 5) not present joint pain, pneumothorax, bronchitis, convulsive crisis, anemia, cold, infection, joint trauma; 6) not have done any diving in the 48 h before the flight simulation and/or dermatological and/or dental treatment; 5) not be a smoker or be using any type of medication. Exclusions criteria were having otalgia, aerocolia, or decompression illness during the analysis of risk factors for middle ear barotrauma. Among the airmen, three were excluded according to the exclusion criteria and the remainder were included in the final analysis (*n* = 23).

### 2.2 Hypobaric flight chamber

Before the flight simulation, participants ate a single standard meal. The flight simulation protocol adopted in the hypobaric chamber was similar to the standard proposed by the Civil Aerospace Medicinal Institute (CAMI) of the Federal Aviation Administration. A hypobaric chamber measuring 7.3 m× 2.7 m× 2.4 m, with a capacity of 16 individuals was used, from the Institute of Aerospace Medicine (IMAE), located in Rio de Janeiro.

**FIGURE 1:**
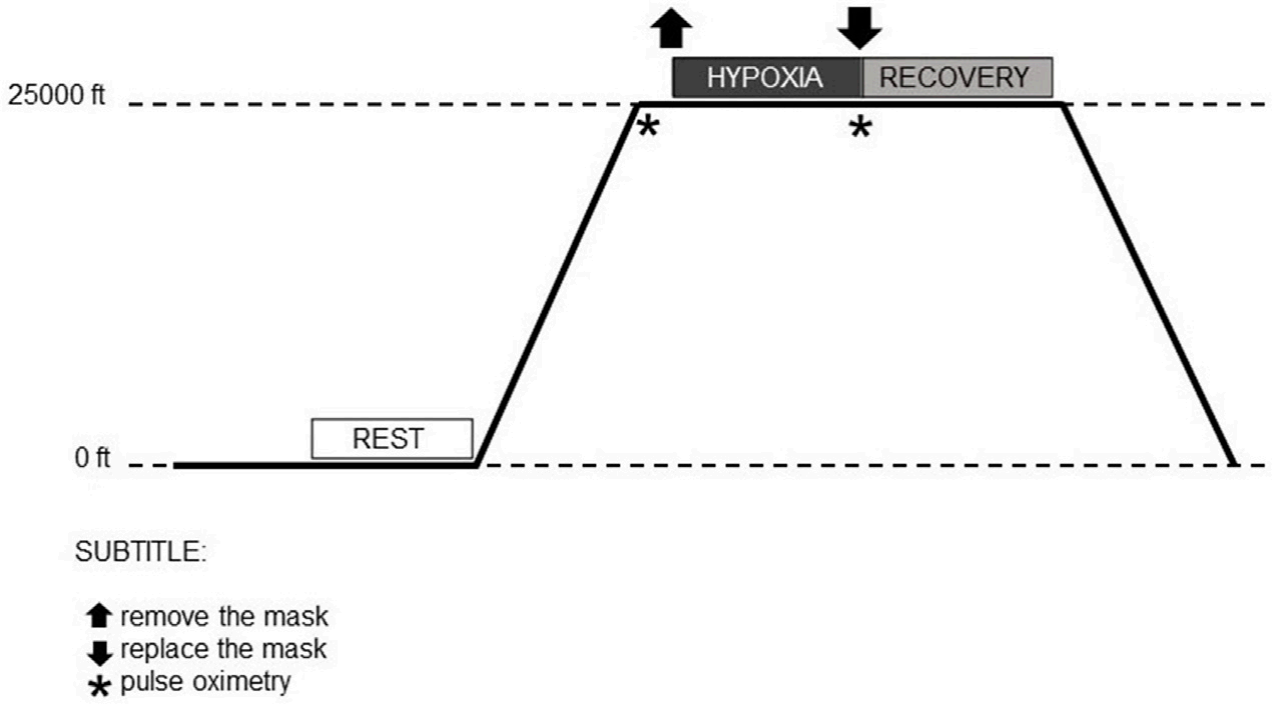
Moments of the flight simulation in the hypobaric chamber.

The flight simulation was divided into three stages: 1) denitrogenation; 2) ascent to 25,000 ft (7620 m) to demonstrate acute hypoxia; and 3) return to sea level, as illustrated in Figure 1.

The airmen remained seated inside the hypobaric chamber, equipped with an aviation helmet (model HGU-55 CE—Gentex Corporation^®^, Inc., PA, United States), naval aviation O_2_ face mask (standard model MBU-12, Gentex Corporation^®^, Inc., PA, United States), heart monitor, and pulse oximeter, together with the medical team. Initially, at sea level, the airmen breathed 100% O_2_ for approximately 30 min. This procedure aims to minimize the risk of decompression sickness and barotrauma due to body nitrogen concentrations.

Subsequently, the hypobaric chamber simulated ascent to an altitude of 25,000 ft (7620 m), at which point the airmen removed the O_2_ face aviation mask and kept it off until the first symptoms of hypoxia were identified (e.g., vision impairment, mental fatigue, shortness of breath, headaches). At the time of exposure to hypobaric hypoxia, the symptoms and their identification were individual. After individual and self-reported recognition of early symptoms (TAS), the airmen resumed O_2_ supplementation (100%) by putting the O_2_ face mask back on and activating the emergency breathing device.

### 2.3 Assessment of cardiac autonomic function-HRV

To acquire the RR intervals (iRR), the Polar Team 2 (Polar Heart Rate Monitor^®^) was used. Before starting the flight simulation, the heart rate monitor belt (Model H10- Polar Electro, Inc, NY, United States) was moistened and positioned on the chest, according to the manufacturer’s instructions. The airmen then remained comfortably seated inside the hypobaric chamber with their eyes open and breathing spontaneously. The iRRs were recorded during the hypobaric chamber flight simulation.

For the analysis of cardiac autonomic behavior, the following moments were considered: Rest: at sea level, in the final 2 min and 30 s of the 30 min during which the airmen breathed 100% O_2_. Hypoxia: at 25,000 ft, the time lapse between the O_2_ mask removal and its replacement after the first individually identified symptoms of hypoxia, with variable time. Recovery: at 25,000 ft, the first 2 min and 30 s from the O_2_ mask replacement. The interval time (2m30s) was the maximum time reached during hypoxia, so, in order to match the analysis times between the moments, this time was chosen for all moments.

The data were instantly transmitted to the computer *via* the Polar Team 2^®^ software. HRV analysis was performed using Kubios software (version 2.2 Biosignal Analysis and Medical Imaging Group, Kuopio, Finland). To correct the artifacts, the evaluators used the Kubios automatic filter (medium error correction). In addition, visual inspection of the graphs was carried out by two experienced researchers.

HRV was analyzed in the time domain, including the following indices: R-R (ms)—Average of R-R intervals in milliseconds. SDNN (ms)—Standard deviation of RR intervals in milliseconds. HR (bpm)—Mean heart rate in beats per minute. RMSSD (ms) square root of successive differences between R-R intervals in milliseconds. Also, in the non-linear domain, including the indices: SD1—The standard deviation of the Poincaré plot perpendicular to the identification line. SD2—The standard deviation of the Poincaré plot along the identification line.

### 2.4 Pulse oximetry analysis

During the entire hypobaric flight simulation, the airmen remained connected to the Mindray PM-60 O_2_ saturation sensors (Mindray Bio-Medical Electronics Co, Shenzhen, China) positioned on the second distal phalanx of the non-dominant hand to verify the saturation percentage. (%S O_2_). O_2_ saturation (S O_2_) measurements were evaluated at rest and at the end of the hypoxic period.

### 2.5 Anthropometric and body composition data

Bodyweight (Filizola^®^, São Paulo, Brazil) and height (Sanny^®^ Stadiometer) were evaluated following previously published methods (Quetelet, 1994; Keys et al., 2014), before the flight simulation in a hypobaric chamber. The body mass index (BMI) was calculated as kg/m^2^ (Quetelet, 1994; Keys et al., 2014). Body fat was estimated using the three skinfold protocol proposed by Jackson and Pollok (Jackson and Pollock, 1978), performed in duplicate using a skinfold caliper (Lange^®^, Cambridge Scientific Instruments, Maryland, United States). Body density was calculated and converted to body fat percentage using the equation reported by Siri (Siri, 1993).

### 2.6 Physical Conditioning Assessment Tests

To verify the physical performance of the airmen, twice a year they are obligatorily submitted to physical conditioning evaluation tests. As the cardiorespiratory fitness assessment and neuromuscular tests (push-ups on the ground and trunk flexion on the thighs) were performed 1 month before (September 2019) the flight simulation, a documentary analysis of the results archieved by the airmen was carried out.

#### 2.6.1 VO_2_ submaximal

The values of VO_2submaximal_ (ml.kg^−1^.min^−1^) were obtained following the recommendations of the Cooper test (Cooper, 1968). Airmen were required to run/walk for 12 min on an official athletics track (400 m), covering the greatest possible distance. The distance traveled in meters was then applied to the equation: VO_2submaximal =_ distance traveled in meters—504.9/44.73 (Cooper, 1968).

#### 2.6.2 Push-ups on the ground

In the ventral decubitus position, with hands supported in front on the floor and the shoulders slightly apart about the projection, keeping the body fully extended. The upper limbs are then flexed so that the back exceeds the line of the elbows, and the elbows are projected out approximately 45° to the trunk. Upon reaching this position, the upper limbs are extended, returning to the starting position. The maximum number of repetitions performed without a Pollock adapted time limit was counted (Michael L. Pollock and Wilmore, 1993; Riebe et al., 2018).

#### 2.6.3 Flexion of the trunk on the thighs

Lying in dorsal decubitus, hands crossed at the chest at shoulder height, knees at a 90° angle, feet aligned and fixed with the help of the evaluator. The trunk is then flexed until the elbows touch the distal third of the thighs, returning to the starting position. The maximum number of repetitions performed in 1 min was counted. (Michael L. Pollock and Wilmore, 1993; Riebe et al., 2018).

### 2.7 Statistical analysis

*A posteriori* sample size of 23 participants provided a statistical power of 93% (1-β = 0.93), for a significance level of 5% (*α* = 0.05) and large effect size (f = 0.80). Data normality was assessed using the Shapiro-Wilk test. The significance level adopted was 5% (*p* < 0.05). The variables related to HRV indices showed non-parametric distribution. To compare the values of heart rate variability indices at rest, during hypoxia and recovery, the Kruskal-Wallis test was applied, followed by Dunn’s post-test. Data are expressed as mean and standard deviation. For the TAS variables, final O_2_ saturation, O_2_ saturation delta, weight (kg), height (m), BMI (kg/m2), waist-to-height ratio (RCE), fat percentage, lean mass percentage, fat in kg, fat-free mass in kg, distance covered, and VO_2submaximal_, a descriptive analysis was performed, and data are expressed as mean and standard deviation. Finally, the association between the variables was performed using the Wilcoxon paired difference test. Statistical analyses were performed using G*Power (v3.1), SPSS 21 (IBM, SPSS Statistics^®^ Inc., Illinois, United States), and GraphPad Prism (v6.0).

## 3 Results

Anthropometric characteristics, body composition, results of physical fitness tests, and O_2_ saturation of the airmen are described in Table 1.

**TABLE 1:** Sample characterization and physical assessment results.

Variables	Mean (*n* = 23)	SD
Anthropometry and body composition		
Age (years)	30.70	±6.70
Body Mass (kg)	83.41	±7.34
Height (m)	1.76	±0.05
Body mass index—BMI (kg/m^2^)	26.91	±2.05
Waist circumference (cm)	87.10	±4.35
Waist-to-height ratio—RCE	0.49	±0.02
Body fat (%)	16.91	±4.55
Fat free mass (%)	83.08	±4.55
Fat mass (kg)	14.18	±4.27
Fat free mass (kg)	69.24	±6.38
Physical fitness tests		
Ground push-up (reps)	38.61	±8.14
Trunk flexion (reps)	49.13	±9.21
Distance covered—Cooper 12’ (m)	2390.52	±244.28
VO_2 submaximal_	41.92	±5.43
O_2_ saturation		
Initial O_2_ saturation (%)	99.61	±0.65
Final O_2_ saturation (%)	80.96	±9.45
O_2_ saturation delta	18.65	±9.44
TAS	1m40s	±28 ms

Among the first symptoms self-reported by the airmen, there was a frequency of 4% euphoria, 4% headache, 9% tingling in the limbs, 26% slow thinking, and 57% vision alteration.

**FIGURE 2:**
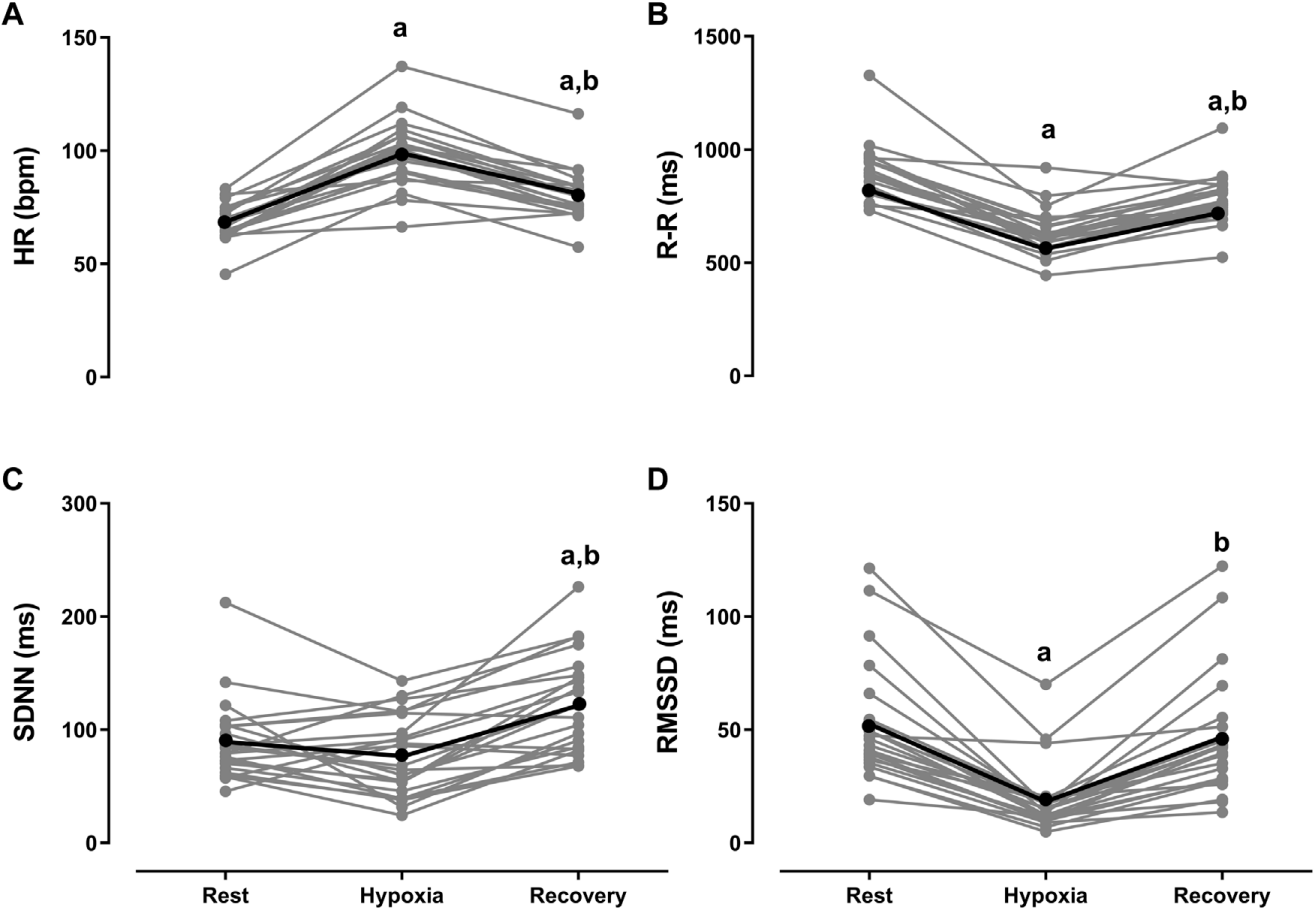
Kruskal-Wallis test followed by Dunn’s post-test for multiple comparisons. Data expressed as mean ± standard deviation. Ms, milliseconds. **(A)** HR (bpm)—mean heart rate in beats per minute. **(B)** R-R (ms)—mean of R-R intervals in milliseconds. **(C)** SDNN (ms)—standard deviation of RR intervals in milliseconds. **(D)** RMSSD (ms) square root of successive differences between R-R intervals in milliseconds. ^a^ *p* < 0.0001 vs rest. ^b^ *p* < 0.0001 vs hypoxia.

Comparison of mean values of HRV indices during rest, hypoxia, and recovery are illustrated in Figure 2.

In the comparison between hypoxia vs resting, the values of iRR, RMSSD, NN50 and SD1 indices decreased (*p* < 0.0001). The SDNN and SD2 indices did not change. In the comparisons between recovery vs resting, the value of the iRR decreased (*p* < 0.0001). The SDNN indices increased (*p* < 0.0001). The RMSSD and SD1 indices did not change in the comparisons between recovery vs hypoxia, the iRR, SDNN, RMSSD, SD1 and SD2 indices increased (*p* < 0.0001), and only the HR decreased (*p* < 0.0001).

Visual and individual analysis of hypoxia-rest variation showed that 100% of the airmen had a negative delta for the mean HR, iRR and RMSSD indices. An example of the graphical behavior of iRR is illustrated in Supplementary Figure S1—SF1. From the visual inspection, it was possible to perceive a decrease in the length and amplitude of the RR intervals and the loss of linearity during hypoxia.

We stratified the airmen into “high-HRV” and “low-HRV” considering the rest HRV values in ascending order and compared the values of HRV indices between these groups at rest, hypoxia, and recovery times. See Table 2.

**TABLE 2:** Heart rate variability during baseline, hypoxia, and recovery.

HRV indices	High			Low			Mann-whitney		
	Mean	SD	Median	Mean	SD	Median	U	Z	*P*
RR (ms)									
Baseline	981.75	117.32	944.18	824.81	55.91	834.84	3.000	−3.877	0.000
Hypoxia	678.06	108.02	658.34	593.26	69.95	611.35	40.000	−1.600	0.118
Recovery	841.77	95.87	824.02	722.82	75.89	728.79	14.000	−3.200	0.001
SDNN (ms)									
Baseline	107.94	43.82	100.78	69.34	15.99	64.84	19.000	−2.893	0.003
Hypoxia	89.80	41.25	96.79	65.55	25.33	59.87	42.000	−1.477	0.151
Recovery	144.23	44.27	146.05	101.76	31.97	87.49	28.000	−2.339	0.019
HR (bpm)									
Baseline	62.66	6.07	64.32	73.61	5.17	72.23	3.000	−3.877	0.000
Hypoxia	92.12	13.19	91.26	103.88	13.68	100.19	41.000	−1.539	0.134
Recovery	74.49	7.37	73.63	86.02	10.84	83.35	18.000	−2.954	0.002
RMSSD (ms)									
Baseline	67.42	29.82	52.25	35.99	8.55	36.05	9.000	−3.508	0.000
Hypoxia	25.69	19.08	17.34	12.63	4.65	12.00	30.000	−2.216	0.027
Recovery	62.98	29.69	51.25	30.38	9.93	30.73	10.000	−3.447	0.000
SD1 (ms)									
Baseline	47.83	21.17	37.04	25.51	6.06	25.55	9.000	−3.508	0.000
Hypoxia	18.30	13.51	12.43	9.01	3.29	8.59	31.000	−2.154	0.032
Recovery	44.67	21.07	36.33	21.55	7.04	21.80	10.000	−3.447	0.000
SD2 (ms)									
Baseline	144.01	59.94	136.68	94.42	22.69	87.19	23.000	−2.646	0.007
Hypoxia	124.38	57.83	135.30	91.48	36.01	84.06	42.000	−1.477	0.151
Recovery	197.64	60.84	201.47	141.38	45.41	120.02	28.000	−2.339	0.019

R-R (ms)—average of R-R intervals in milliseconds, SDNN (ms)—standard deviation of RR, intervals in milliseconds, HR (bpm)—mean heart rate in beats per minute, RMSSD (ms) square root of successive differences between R-R intervals in milliseconds, SD1—The standard deviation of the Poincaré plot perpendicular to the identification line, SD2—The standard deviation of the Poincaré plot along the identification line.

At rest and recovery times, the cardiac autonomic behavior of airmen classified as “high-HRV” differed from those who presented “low-HRV” (*p* < 0.000), except for the SD-HR index. During hypoxia, the iRR (*p* = 0.118), SDNN (*p* = 0.151), Mean HR (*p* = 0.134), and SD2 (*p* = 0.151) indices did not differ between airmen classified as high-HRV when compared to airmen with low-HRV. However, for the representative indices of the parasympathetic activity, RMSSD (*p* = 0.027), NN50 (*p* = 0.044), and SD1 (*p* = 0.032) the airmen classified as “high-HRV” presented higher values when compared to the airmen classified as ‟low-HRV”.

The results of the Spearman correlation between HRV indices at different moments of flight (i.e., rest, hypoxia, and recovery) and the results of physical fitness tests are described in Table 3.

**TABLE 3:** Spearman correlations between HRV indices at different moments and VO_2_ estimated, trunk flexion (reps), and push-ups on the ground (reps).

HRV indexes	VO_2_ estimated			Trunk flexion (reps)			Push-up on the ground (reps)		
	Baseline	Hypoxia	Recovery	Baseline	Hypoxia	Recovery	Baseline	Hypoxia	Recovery
RR (ms)	0.239	0.326	0.312	0.486	0.414	0.434	0.168	0.050	0.122
SDNN (ms)	0.318	0.229	0.441	0.466	0.359	0.417	0.315	0.380	0.377
HR (bpm)	−0.223	−0.305	−0.256	−0.466	−0.358	−0.402	−0.162	0.006	−0.064
RMSSD (ms)	0.300	0.424	0.371	0.492	0.443	0.463	0.371	0.246	0.289
SD1 (ms)	0.300	0.433	0.371	0.492	0.435	0.463	0.371	0.246	0.289
SD2 (ms)	0.274	0.216	0.449	0.431	0.355	0.394	0.266	0.375	0.377

R-R (ms)—Average of R-R intervals in milliseconds. SDNN (ms)—standard deviation of RR, intervals in milliseconds. HR (bpm)—mean heart rate in beats per minute. RMSSD (ms) square root of successive differences between R-R intervals in milliseconds. SD1—The standard deviation of the Poincaré plot perpendicular to the identification line. SD2—The standard deviation of the Poincaré plot along the identification line.

Regarding the estimated VO_2_: 1) at rest there was no association; 2) during hypoxia there was an association with the RMSSD and SD1 indices (parasympathetic markers); and 3) in recovery there was an association with the SDNN and SD2 indices (sympathetic markers). Regarding the trunk flexion test: 1) the moment of rest was associated with the Mean RR, SDNN, Mean HR, RMSSD, SD1, and SD2 indices; 2) during hypoxia there was an association with the Mean HR, RMSSD, NN50, and SD1 indices; and 3) in recovery there was an association with the Mean RR, SDNN, Mean HR, RMSSD, and SD1 indices. There was no association between HRV indices and the results of the push-ups on the ground. Therefore, airmen who showed parasympathetic predominance suffered less interference from HH and, therefore, seemed to deal better with this physiological stress.

The Spearman’s correlation between TAS (seconds) and body composition variables, and the results of physical fitness tests, O_2_ saturation, and HRV indices at rest showed no association between the variables.

## 4 Discussion

To the best of our knowledge, this is the first study to analyze the influence of HH on cardiac autonomic function during TAS and its association with indices of physical fitness in airmen. Our main findings were: 1) HH induced a decrease in the values of the HRV indices of the airmen during the TAS; 2) the rMSSD index was the most sensitive to changes induced by HH; 3) airmen with high-HRV showed parasympathetic dominance during hypoxia; 4) the results of the physical fitness tests were associated with the HRV indices during the moments of the flight; 5) the HRV indices and the results of the physical fitness tests (TACF) did not present a linear correlation with the TAS.

The results of the present study demonstrated a decrease in iRR during TAS, even though its time-lapse was shorter than that of TUC. That is, cardiac autonomic modulation presented similar behavior in both situations. In previous studies, airmen were exposed to HH, on average, for 3 min and 32 s (Barak et al., 1999; Yoneda et al., 2000; Guadagno et al., 2011). In the present study, airmen were exposed to HH for an average of 1 min and 47 s. However, considering only the HRV, the iRR decrease occurred quickly, even before the self-report of the physiological symptoms of HH (TAS), thus minimizing the risks to the physical and mental integrity of the airmen, as well as the risks to flight safety.

In the visual inspection of the airmen’s iRR, a decrease in the length and amplitude of the RR intervals and loss of linearity during hypoxia were observed (Supplementary figure). The scientific literature presents a graphic illustration reporting iRR behavior similar to what we saw in the present study. However, the studies differ in the flight simulation protocol regarding altitude (25,000 vs 27,000 ft) and HH exposure time (TAS vs TUC), but together this evidence indicates a reduction in HRV(Barak et al., 1999; Vigo et al., 2010).

**FIGURE 3:**
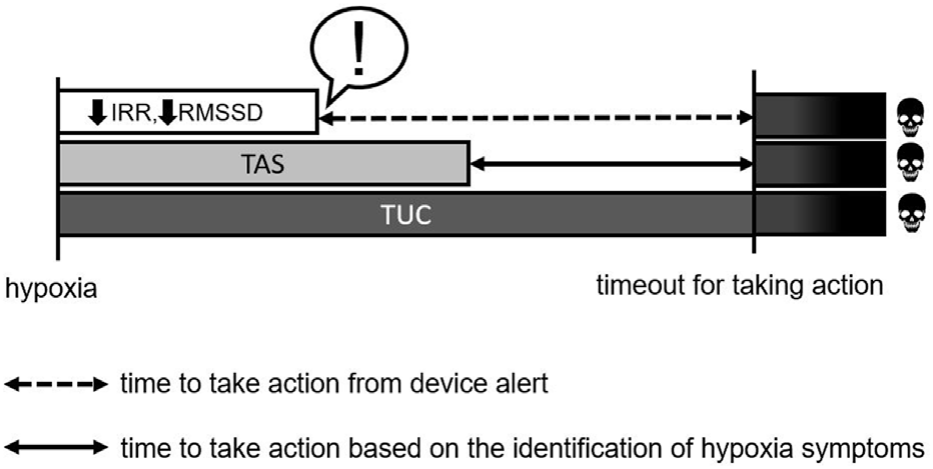
R-R (ms)—average of R-R intervals in milliseconds; RMSSD (ms) square root of successive differences between R-R intervals in milliseconds; TAS-time of onset of symptoms; TUC-useful time of consciousness. !—Alert.

Within the first few minutes of exposure to HH at 25,000 ft, the decrease in HRV indices is noticeable. Therefore, there is no need to expose airmen to HH for a long time to identify the physiological symptoms of HH. The analysis of the change in HRV can contribute to the creation of warning devices for airmen (Mellor et al., 2018; Rice et al., 2019). Considering that the time factor in an emergency situation such as HH can be decisive for the occurrence or not of a fatality, the use of a device like this will help to guarantee the maintenance of the physical integrity of the airman and, therefore, the safety of the flight, through an “early” alert. See Figure 3.

It is important to note that among the indices evaluated, the two most sensitive for quantifying cardiac autonomic modulation in HH, paired by exposure time, and were iRR and RMSSD, since in the recovery-hypoxia variation all airmen had a negative delta. Thus, it is suggested that the iRR and RMSSD indices be used as a reference for the creation of a mathematical equation that alerts to the occurrence of HH as soon as possible. However, knowing the complexity of the interaction of physiological systems, multiple factors need to be considered to create this equation, such as: altitude and barometric pressure. In addition, considering the need to evaluate its validity, accuracy, and reproducibility, more accurate analyses and the evaluation of direct measurements and re-tests are necessary.

Another important physiological measure for this equation is peripheral arterial O_2_ saturation, which is a useful marker to identify physiological changes related to high altitude, as its values decrease with a drop in atmospheric pressure (Barak et al., 1999; Saito et al., 2005; Wolf and Garner, 2009; Vigo et al., 2010). In the present study, even though the exposure time to HH was shorter, we identified a drop in O_2_ saturation, corroborating the findings of previous studies (Hoffman et al., 1946; Barak et al., 1999; Yoneda et al., 2000; Vigo et al., 2010; Guadagno et al., 2011). However, the O_2_ saturation delta of the airmen in the present study was lower than the studies presented in Table 4 and Table 5. In this sense, the evidence shows that the effects of HH depend on the severity of the hypoxia relative to 1) the exposure time (Barak et al., 1999; Guadagno et al., 2011), 2) the magnitude of the altitude (Hoffman et al., 1946; Vigo et al., 2010); and 3) the individual (Temporal, 2005; Russomano, 2012; de Carvalho, 2015; Russomano and Castro, 2020).

**TABLE 4:** Spearman correlation between TAS (seconds) and body composition variables, physical fitness test results, oxygen saturation, and resting HRV indices.

Variables	*R* ^2^	r	p
Body composition			
Weight (kg)	0.0324	−0.18	0.42
waist-to-height ratio	0.0016	−0.04	0.85
Body fat (%)	0.0169	−0.13	0.57
Fat free mass (%)	0.0169	0.13	0.57
Physical fitness indices			
Ground push-up (reps)	0.0324	0.18	0.41
Trunk flexion (reps)	0.0576	0.24	0.26
VO_2 submaximal_	0.0256	0.16	0.47
Final O_2_ saturation	0.0144	−0.12	0.6
O_2_ saturation delta	0.0225	0.15	0.5
Resting heart rate variability indices			
RR (ms)	0.0081	0.09	0.70
SDNN (ms)	0.0016	0.04	0.84
HR (bpm)	0.0081	−0.09	0.69
RMSSD (ms)	0.0225	−0.15	0.502
SD1 (ms)	0.0225	−0.15	0.502
SD2 (ms)	0.0036	0.06	0.78

R-R (ms)—average of R-R intervals in milliseconds. SDNN (ms)—standard deviation of RR, intervals in milliseconds. HR (bpm)—mean heart rate in beats per minute. RMSSD (ms) square root of successive differences between R-R intervals in milliseconds. SD1—The standard deviation of the Poincaré plot perpendicular to the identification line. SD2—The standard deviation of the Poincaré plot along the identification line.

**TABLE 5:** Relationship between altitude and average time of exposure to HH and SO_2_.

Authors	Sample	Altitude	Exposure time average	∆SO_2_
Present study	23	25,000 ft	1 min and 47 s	**−**19
Guadagno et al. (2011)	4	25,000 ft	2–5 min	**−**37.4
Yoneda et al. (2000)	369(26.8 years)	25,000 ft	3 min and 57 s	**−**41.6
	160(45.1 years)	25,000 ft	3 min and 21 s	**−**35.5
Barak et al. (1999)	21	25,000 ft	4 min and 40 s	**−**53
Vigo et al. (2010)	12	27,000 ft	1 min and 10 s	**−**33.5
Hoffman et al. (1946)	25	28,000 ft	2 min and 21 s	**−**54
	25	30,000 ft	1 min and 38 s	**−**54
	25	35,000 ft	1 min and 12 s	**−**58
	25	38,000 ft	47 s	**−**57

Thus, the increase in heart rate, the sharp drop in O_2_ saturation, and the appearance of the first subjective symptoms reinforce the idea that the observed changes were caused by hypoxemia and give indications of the manifestation and evolution of the first stages of hypoxia at 25,000 ft (Yoneda et al., 2000; Temporal, 2005; Russomano and Castro, 2020) although the time of exposure to HH was shorter (TAS vs TUC). See Figure 3.

Initially, we hypothesized that the physical fitness and HRV indices of the airmen would be associated with the TAS, so that individuals with a better level of physical fitness and, therefore, higher values of the HRV indices, would have longer TAS, which may resemble the time-lapse of the TUC, without, however, approaching loss of consciousness. Although this association was also considered by other researchers (Barak et al., 1999), to date it has not yet been researched. However, contrary to the initial hypothesis, physical fitness and HRV indices were not associated with TAS.

In this perspective, it is important to point out that, over the years, different methods have been used to define the criterion that delimits the “endpoint” of the time of exposure to hypoxia (Izraeli et al., 1988; Yoneda et al., 2000). This fact makes comparisons between studies difficult. Furthermore, to the best of our knowledge, this is the first study to use the TAS instead of the TUC.

Another important aspect lies in the TAS/TUC evaluation criteria, based on self-reported subjective perception, which in turn, can suffer various interferences, as airmen are placed in an environment that induces changes in cognitive function (Izraeli et al., 1988; Yoneda et al., 2000). Thus, the perception of the first symptoms could be wrong. As an example, it has been suggested that the indication of TUC may be influenced by the tendency of novice airmen not to report the symptoms due to a possible feeling of competitiveness and/or the veterans’ experience in recognizing HH symptoms more quickly (Yoneda et al., 2000). Despite the aforementioned limitations, it is possible that in the present study this measure does not reflect the actual moment of onset of the first symptoms.

In this sense, a study showed that smokers, that is, with increased levels of carboxyhemoglobin (responsible for inducing anemic hypoxia) had self-reported subjective symptoms and TUC similar to non-smokers (Yoneda and Watanabe, 1997). Furthermore, it has been suggested that genetic polymorphism (mitochondrial-DNA—haplogroup D) may influence hypoxia tolerance time (Motoi et al., 2016). In addition, the predominance of muscle fiber type seems to influence hypoxia tolerance. Type I muscle fibers are more resistant to the effects of hypobaric hypoxia (Chaudhary et al., 2012).

Sympathetic hyperreactivity and/or maintenance of the fight-or-flight state (sympathetic predominance) during rest cause damage to tissue and, consequently, to health (Rakhshan et al., 2015; Sharma, 2018). Several cardiovascular diseases are associated with hyperreactivity (Fisher and Paton, 2012; Karia et al., 2012; Carter and Ray, 2015). In this sense, a recent study carried out with an animal model reported that intermittent hypoxia generates systemic and cardiac sympathoactivation and is an important risk factor for sudden cardiac death after cardiac ischemia (Morand et al., 2018). On the other hand, physical exercise contributes to a reduction in sympathetic activation and an increase in cardiac vagal modulation, promoting an antiarrhythmic effect (Soares-Miranda et al., 2009; Carter and Ray, 2015). Additionally, the level of physical fitness seems to influence sympathetic reactivity (Ifuku et al., 2007) and positively regulate the autonomic balance (Kwon et al., 2014; Carter and Ray, 2015; Fisher et al., 2015), and physical training is the best agent to improve physical fitness, by improving cardiac efficiency and O_2_ extraction by peripheral tissues (Saltin, 1968). Therefore, effective/efficient cardiac autonomic balance is essential for maintaining health in hypoxic situations. The aforementioned reports corroborate the results of the present study in which the physically trained airmen showed an adequate autonomic response to HH. Furthermore, those classified with high-HRV had higher values of the representative indices of the parasympathetic branch (RMSSD, NN50, and SD1) when compared to airmen classified as “low-HRV” during hypoxia and recovery.

Furthermore, after a stressful event, such as HH, it is of paramount importance that airmen can quickly reestablish physiological homeostasis and resume cognitive, psychomotor, piloting, and flight performance, and flight safety (Files et al., 2005; Steinman et al., 2017; Rice et al., 2019). Nevertheless, cardiac autonomic recovery has been considered an important mortality rate and can be influenced by the level of physical fitness and physical training (de Oliveira and de Lima, 2012).

Finally, for better understanding, we emphasize that the physiological complexity and its adaptations to the hypobaric hypoxic environment require more sophisticated and accurate methods. However, this methodological robustness and its reproduction are too expensive and access to the hypobaric chamber for simulating flights at high altitudes for hypoxia simulation is extremely restricted.

Despite planning and efforts to prevent limitations, we recognize that there are opportunities for improvement in future investigations. First, it was not possible to strictly control the airman’s power supply. However, the only meal before the flight simulation in HH was standard for all participants. Second, the lack of individuals with different levels of physical fitness and a control group as access to the flight simulation in HH is extremely restricted and too expensive, preventing testing with different people. Despite this, the present study provides evidence of HRV assessed by a cardiac monitor as a promising tool for assessing cardiac autonomic modulation in HH at 25.000 ft.

In summary, we concluded that HH induced cardiac autonomic changes which could be identified through the cardiac monitor *Polar Team* ^®^ even before airmen self-reported the first subjective symptoms. This demonstrates that cardiac autonomic modulation appears to be more sensitive to the effects of HH at 25.000 ft than the self-reported subjective perception of symptoms. Therefore, portable devices capable of providing an early warning of the occurrence of hypobaric hypoxia could be created from the HRV, buying the airman more time to take action to re-establish control and flight safety.

## Data Availability

The raw data supporting the conclusions of this article will be made available by the authors, without undue reservation.

## Supplementary material

Supplementary Figure S1 An example of the graphical behavior of R-R. ms, milliseconds.
