# Supplementary figures and images for: Could cardiac autonomic modulation be an objective method to identify hypobaric hypoxia symptoms at 25.000ft among Brazilian military airmen?
Source: Front Physiol. 2022 Nov 3;13:1005016. doi: 10.3389/fphys.2022.1005016 (PMC9669981; doi:10.3389/fphys.2022.1005016)

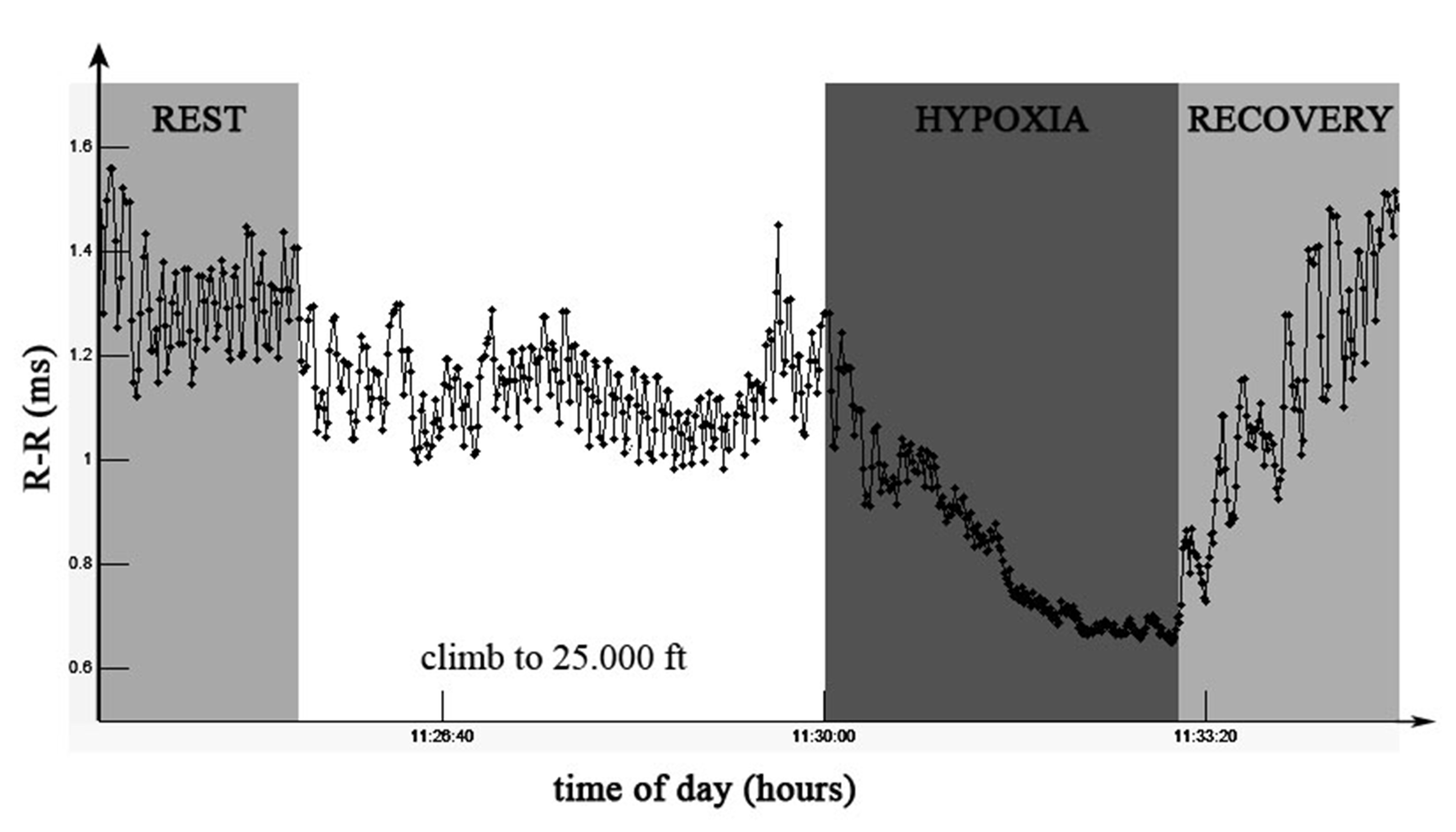

Supplement: Supplementary file 1 [file Image1.JPEG]
